# Intraductal tubular adenomas (pyloric gland-type) of the pancreas: clinicopathologic features are similar to gastric-type intraductal papillary mucinous neoplasms and different from intraductal tubulopapillary neoplasms

**DOI:** 10.1186/s13000-014-0172-1

**Published:** 2014-09-23

**Authors:** Xiaoyan Chang, Ying Jiang, Ji Li, Jie Chen

**Affiliations:** Department of Pathology, Peking Union Medical College Hospital, Chinese Academy of Medical Sciences and Peking Union Medical College, Tsinghua University, Beijing, 100730 PR China

**Keywords:** Pancreas, Intraductal tubular adenoma, Pyloric gland adenoma, Intraductal papillary mucinous neoplasm, Intraductal tubulopapillary neoplasm

## Abstract

**Background:**

Intraductal tubular adenoma of the pancreas, pyloric gland type (ITA), is an infrequent intraductal benign lesion located in the main duct and large branch duct of the pancreas. The purpose of this report is to introduce seven new cases and to compare their clinicopathologic features and KRAS mutations to gastric-type intraductal papillary mucinous neoplasms (IPMNs) and intraductal tubulopapillary neoplasms (ITPNs).

**Methods:**

Clinical findings, morphologic features, immunophenotypes and KRAS alterations were investigated in 7 patients with intraductal tubular adenomas, 16 patients with gastric-type intraductal papillary mucinous neoplasms and 6 patients with intraductal tubulopapillary neoplasms.

**Results:**

There were more female patients in the ITA and gastric-type IPMN groups, whereas the opposite pattern was observed in the ITPN group. ITAs and gastric-type IPMNs were lined by columnar cells, similar to pyloric glands, with large extracellular deposits of mucin. ITPNs were polypoid and papillary mass located in the pancreatic ducts, which did not show large deposits of mucin. All ITAs and gastric-type IPMNs expressed MUC5AC strongly and diffusely, and 3/6 ITPNs expressed MUC5AC focally and weakly. KRAS mutations were identified in 4 ITAs (4/7, 57%), 9 IPMNs (9/16, 56%) and 2 ITPNs (2/6, 33%).

**Conclusion:**

The intraductal tubular adenoma should not be considered a precursor lesion of intraductal tubulopapillary neoplasms. No adequate data established ITA should separate as a specific entity from IPMNs.

**Virtual Slides:**

The virtual slide(s) for this article can be found here: http://www.diagnosticpathology.diagnomx.eu/vs/13000_2014_172

## Background

Due to the application of advanced medical imaging, more and more intraductal lesions of the pancreas are being detected; however, their pathological classification is more complex. The term of intraductal papillary mucinous neoplasm (IPMN) has traditionally been used to describe them, and it is widely recognized. However, some other parallel nomenclatures also exist. In the 2010 edited WHO classification of digestive diseases, intraductal lesions of the pancreas were divided into two types: IPMNs and a new entity termed intraductal tubulopapillary neoplasms (ITPNs) [1].

In 1999, the term ‘pyloric gland adenoma’ was first put forward by Bakotic B.W. as a name for a novel pancreatic intraductal lesion that was distinct from IPMN [2]. Subsequently, 17 cases have been documented in English literature [2-10] and have been given the name ‘intraductal tubular adenoma’ (pyloric gland-type; ITA). ITAs showed some similarities with IPMNs and ITPNs and also some obvious differences from them.

Besides the WHO classification of intraductal lesions, another system classified intraductal tumors into IPMNs and intraductal tubular neoplasms (ITNs) based on the papillary or tubular structures [11,12]. ITNs were further subclassified into ITAs and intraductal tubular carcinoma (ITCs) depending on the degree of epithelial dysplasia. In this classification, ITA was a precursor lesion to ITC [3,4]. ITC is regarded as a variant of ITPN according to the tubular architectures. Morphologically, ITAs were the benign form and ITPNs were the malignant form. It is doubtful that ITAs may be the precancerous lesion of ITPNs.

The purpose of this study was to report upon a further 7 cases of ITA and to delineate the clinicopathologic characteristics, immunohistochemical features and KRAS mutation rate in these ITAs compared with IPMNs and ITPNs.

## Methods

### Patients and tissue samples

All selected cases were from Peking Union Medical College Hospital (PUMCH) in 2001–2009 and re-examined by other two senior pathologists. Sixteen cases of gastric-type IPMNs, six cases of ITPNs and seven cases of ITAs were selected. ITAs were diagnosed based on the following definition: a localized polypoid mass within large duct and characterized microscopically by close packing of the tubular pyloric glands. Immunohistochemical staining was performed using the enVision method. All ITA, gastric-type IPMN and ITPN cases were stained for MUC1 (Novocastra Laboratories Ltd., clone Ma695, dilution 1:100), MUC2 (Novocastra Laboratories Ltd., clone Ccp58, dilution 1:100) and MUC5AC (Novocastra Laboratories Ltd., clone CLH2, dilution 1:100). Ki-67 (Immunotech S.A., 1:200) and p53 (Novo, DO7, 1:200) staining was also performed. This study was approved by the Ethics Committee of the Peking Union Medical College Hospital, and informed consent was obtained from all cases.

### DNA samples and mutation analyses

Paraffin-embedded tumor samples were microdissected by hand. Genomic DNA was extracted using QIAmp DNA Mini Kit (Qiagen, Germany). KRAS (exons 12, 13) mutations were detected using two methods: real-time PCR and traditional PCR amplification of genomic DNA and direct sequencing of subsequent PCR products. Real-time PCR was performed using ABI 7500 and StepOne (Applied Biosystems, USA). The PCR reaction mixture was generated based on standard assay procedures. The thermal cycling was as follows: an initial heating step at 95°C for 5 min, followed by 40 cycles of 95°C for 15 sec, 69°C for 10 sec, and 62°C for 60 sec (fluorescence collection). Genomic DNA (40 ng per sample) was amplified using primers covering the coding region. Prior to sequencing, all PCR products were purified (QIAquick PCR Purification Kit; Qiagen). Sequencing was performed by Sangon Corp. (Beijing Sangon, China) using the ABI PRISM 3730XL system (Applied Biosystems, USA). All samples with a genetic alteration in the target gene were subsequently sequenced in the reverse direction to confirm the mutation. Surrounding non-tumorous tissue or matched normal tissue were served as the control.

## Results

### Clinical presentations

The major clinicopathologic features of ITAs, gastric-type IPMNs and ITPNs are summarized in Table 1. More female patients were present in the ITA and gastric-type IPMN groups, whereas the opposite pattern was observed in the ITPN group. The average age of patients was similar. Most patients complained of abdominal discomfort. Some patients found the pancreatic mass by routine checkup. Some patients of the three groups had a history of chronic pancreatitis and diabetes mellitus. CA19-9 and CEA were occasionally elevated. Comparing with gastric-type IPMNs and ITPNs, the patients of ITAs did not have specific clinical presentations. Computed tomography and B ultrasound revealed pancreatic cystic masses, and the head of pancreas was the most frequently involved site for all three lesions. The common bile duct appeared normal.

**Table 1:** **The clinical presentations and pathological features of three intraductal neoplasms of pancreas**

	**Gastric-** **type IPMN**	**ITA**	**ITPN**
Gender (M/F)	10/6	5/2	2/4
Age (average age)	39-78 (61)	47-74 (58)	48-70 (64)
Site (head/body/tail)	10/3/3	4/3/0	4/1/1
**Clinical features**			
Symptoms^※^	8/5/3	4/2/1 (back pain)	4/1/1 (jaundice)
Chronic pancreatitis	5/16	4/7	2/6
Diabetes mellitus	4/16	1/7	3/6
Chronic use of tobacco	6/16	3/7	2/6
CA-199 elevated in blood	5/16	1/7	2/6
CEA elevated in blood	2/16	0/7	0/6
**Pathological features**			
Diameter	1-6 cm	0.6-3 cm	1.5-4.5 cm
Microscopic features	Papillary growth with large mucin	Tubulopapillary growth with mucin	Tubulopapillary growth without luminal mucin
**Immunohistochemistry**			
MUC5AC	16/16	7/7	3/6
MUC1	0/16	0/7	3/6
MUC2	9/16 (goblet cells)	3/7 (goblet cells)	0
Ki-67 index	<1%	2/7 3-5%	>20%
P53	-	-	3/6
KRAS mutation	9/16 (56%)	4/7 (57%)	2/6 (33%)

^※^From left to right: abdominal discomfort/routine checkup/other symptoms, such as jaundice, back pain, et al.

### Pathological findings

**Figure 1:**
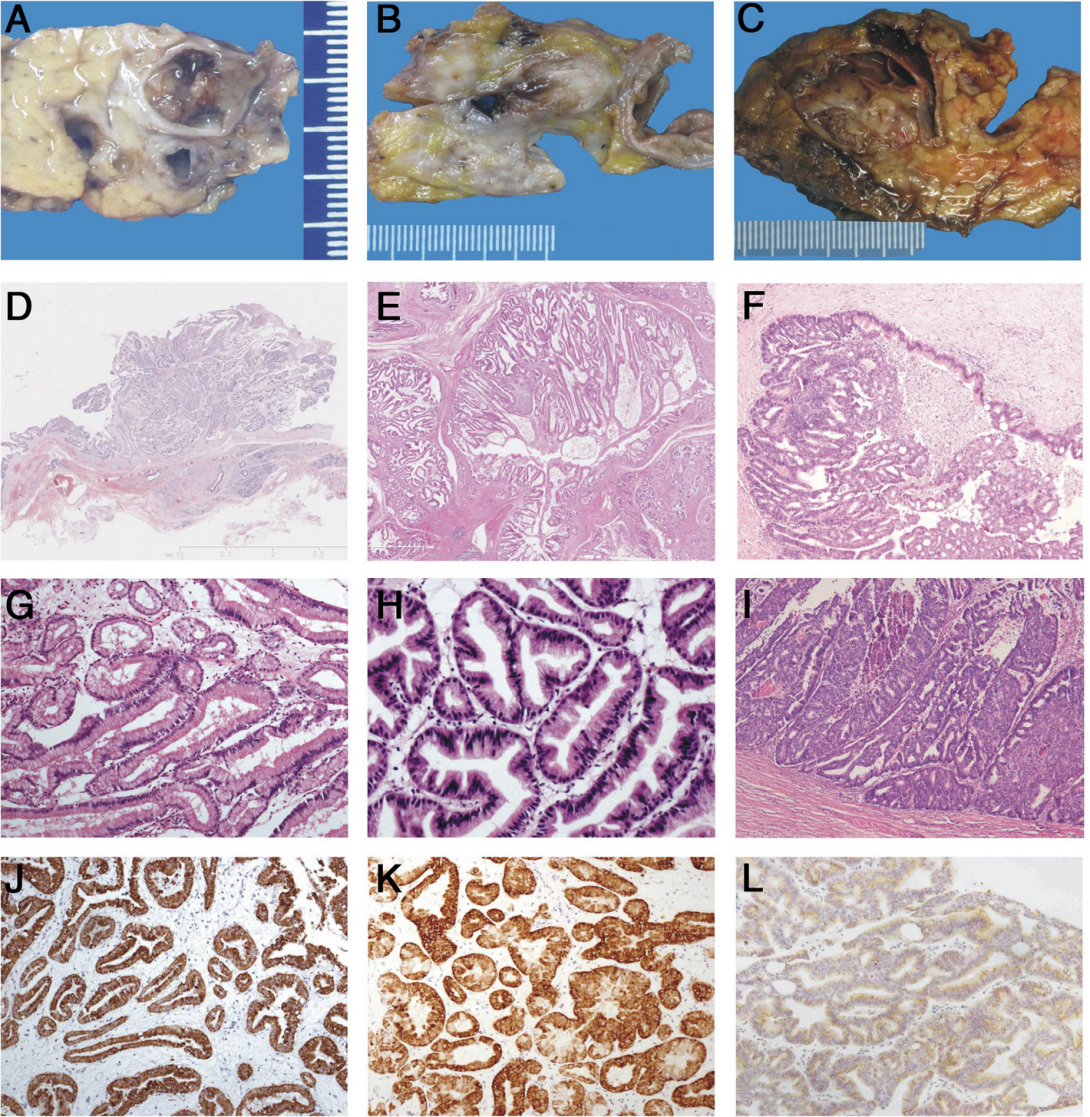
**Pathological comparisons between intraductal tubular adenoma (pyloric gland type; A,D,G,J), gastric-type intraductal papillary mucinous neoplasm (IPMN; B, E, H, K) and intraductal tubulopapillary neoplasm (ITPN; C, F, I, L).** Grossly, ITA and ITPN show a polypoid and nodular mass in the dilated pancreatic duct **(A, C)**, whereas IPMN shows a papillary mass in the mucin-filled dilated duct **(B)**. Microscopically, ITA and IPMN comprise closely packed ducts or tubular glands that are lined with cuboidal-to-columnar mucin-secreting cells with abundant cytoplasm and basally oriented nuclei **(D, E, G, H)**. ITPN shows tightly-packed small glands with a tubulopapillary growth pattern without secreted mucin **(F)**. The neoplastic cells show high-grade atypia with scant cytoplasmic mucin. Intraductal necrotic foci are observed **(I)**. ITA and IPMN express MUC5AC robustly and diffusely **(J, K)** and ITPN expresses MUC1.

All seven ITAs were well demarcated, and polypoids were located within the cystically dilated ducts (Figure 1A, D), which comprised closely packed ducts or tubular glands that resembled pyloric glands (Figure 1G). Gastric-type IPMNs were papillary and mucin was located in the main and branch ducts (Figure 1B, E). The glands were lined with cuboidal to columnar mucin-secreting cells with abundant cytoplasm and basally oriented nuclei similar to ITAs (Figure 1H). Sporadic goblet cells were observed in some ITAs and gastric-type IPMNs. Mild cell atypia was observed with no obvious hyperchromatic nuclei. Mitotic figures were seldom occurred.

Grossly, ITPNs were located in the large pancreatic ducts and showed polypoid nodules that obstructed the duct (Figure 1C, F); a solid nodule was noted beside the dilated duct in one case. Microscopically, ITPNs were characterized by a tubulopapillary growth pattern without secreted mucin and by back-to-back tubular glands (Figure 1I). The neoplastic cells showed high-grade atypia with scant cytoplasmic mucin. Intraductal necrotic foci were observed frequently.

### Immunohistochemical findings

All the MUC expression is summarized in Table 1. ITAs and gastric-type IPMNs expressed MUC5AC (Figure 1J, K) but not MUC1 or MUC2, with negative P53 expression and low proliferative index of ki-67. ITPNs were inclined to expresse MUC1 (Figure 1L) and P53, but not MUC5AC and MUC2, with a high proliferative index.

### KRAS mutations

KRAS mutations were identified in 4 ITAs (4/7, 57%), 9 IPMNs (9/16, 56%) and 2 ITPNs (2/6, 33%). These mutations all showed a single amino acid substitution in codon 12: Gly12Asp (GGT > GAT) or Gly12Val (GGT > GTT), which are the most common mutational foci in pancreatic carcinomas.

## Discussion

IPMN is histopathologically subclassified into four subtypes: gastric, intestinal, pancreatobiliary and oncocytic [1]. The majority of gastric-type IPMNs are IPMNs with low-grade dysplasia (IPMN adenoma). There are some similarities that exist between gastric-type IPMNs and ITAs. They are both located in the pancreatic ducts, producing extracellular mucin and causing marked dilation of the ducts. The lining epithelium comprises columnar cells with morphologic, histochemical and immunohistochemical features similar to those of gastric pylorus, immunoreactive to MUC5AC and negative to MUC1 and MUC2. With regard to behavior, although very few gastric-type IPMNs show high grade atypia or even invasion, gastric-type IPMNs and ITAs are indolent lesions and have a good prognosis.

Kato et al. [4] suggested that ITA was superimposed upon gastric-type IPMN. In 2009, Runjan Chetty and Stefano Serra [6] reviewed all ITA-associated literature and divided ITA into two types, ITA without IPMN (classic ITA or type A) or with gastric-type IPMN (mixed ITA or type B). Fifty percent of the cases reported in the literature may be classified as type A. Some experts have suggested that type A ITA originated from a small focus of gastric/pyloric metaplasia of the ductal cells and that growed into the lumen with no radial extension. However, the classification of ITA had little clinical value. In view of this, even if ITAs were diagnosed as gastric-type IPMN, it would make no difference on clinical therapy.

ITPN is an additional intraductal lesion defined as an intraductal tubule-forming epithelial neoplasm with high-grade dysplasia and ductal differentiation without overt production of mucin [1,13-15]. ITA shows some similarities to ITPN in its histological growth pattern – a tubular pattern with tightly packed small acinar glands. However, from the Table 1, we can see that ITAs were inclined to present in aged men, immunoreactive to MUC5AC and negative for MUC1, whereas ITPNs were inclined to present in aged women,partially negative to MUC5AC and positive for MUC1. The differences in patient population and mucin expression indicate that ITAs and ITPNs are distinct intraductal neoplasms of the pancreas.

Mutations in KRAS exon 2 (codons 12 and 13) are the most frequently detected and earliest event in pancreatic carcinogenesis. The presence of an activating KRAS mutation has been noted in >90% of pancreatic ductal adenocarcinomas (PDACs), and it is not specific for pancreatic carcinoma. In some pre-neoplastic lesions including pancreatic intraepithelial neoplasms (PanINs) and IPMNs, KRAS mutations are detected frequently, increasingly together with cell atypia [16]. In a review of the literature, two cases of ITA showed mutations in KRAS codon 12 [2,4]. In our study the ratio of KRAS mutations in ITAs was unexpectedly high, up to 57%, a rate similar to gastric-type IPMNs (56%) and much higher than that of ITPNs (33%). In general, malignant lesions show higher rates of mutation of this gene than precursor lesions, for example, PanIN3 shows a much greater rate of KRAS mutation than PanIN1. The rate of KRAS mutations does not support the theory that ITAs are a precursor lesion of ITPNs. Toru Furukawa has verified this conclusion atmolecular level [17].

## Conclusions

Intraductal tubular adenoma (ITA) should not be a precursor lesion of ITPN. Up to now, no adequate data established ITA should separate as a specific entity from IPMNs.
